# Association of Vitamin D Status with Radiographic Changes in Knee Osteoarthritis Patients

**DOI:** 10.7759/cureus.103235

**Published:** 2026-02-08

**Authors:** Shamim Farhad, Mohammad Muhibbur Rahman, Shahina Sarker, A.B.M. Mehedi, Md. Obaidul Alam, Fatima Tuz Zahra, Mohammed Emran

**Affiliations:** 1 Physical Medicine and Rehabilitation, Government Employee Hospital, Dhaka, BGD; 2 Physical Medicine and Rehabilitation, Savar Upazilla Health Complex, Savar, BGD; 3 Physical Medicine and Rehabilitation, Directorate General of Health Services, Dhaka, BGD; 4 Physical Medicine and Rehabilitation, Rangpur Medical College and Hospital, Rangpur, BGD; 5 Dermatology, Bangladesh Medical University, Dhaka, BGD; 6 Physical Medicine and Rehabilitation, Khwaja Yunus Ali Medical College, Sirajganj, BGD

**Keywords:** kellgren-lawrence grading system, knee osteoarthritis, radiographic severity, serum vitamin d, vitamin d deficiency

## Abstract

Background: Knee osteoarthritis (KOA) is a common degenerative joint disease and a major cause of pain and disability among older adults. Vitamin D plays an important role in musculoskeletal function, yet its association with radiographic severity of KOA remains unclear, particularly in South Asian populations where deficiency is widespread. This study aimed to assess the relationship between serum vitamin D levels and radiographic KOA among patients attending a tertiary care center in Bangladesh.

Methods: A cross-sectional analytical study was conducted at the Department of Physical Medicine and Rehabilitation at Bangladesh Medical University (BMU), Dhaka, Bangladesh. Patients aged ≥50 years presenting with knee pain were screened, and 90 individuals fulfilling the American College of Rheumatology (ACR) criteria for primary KOA were enrolled through purposive sampling. Weight-bearing anteroposterior and lateral knee radiographs were obtained and graded using the Kellgren-Lawrence (K-L) classification. Serum 25-hydroxyvitamin D levels were measured using the Abbott Architect chemiluminescent microparticle immunoassay (CMIA) assay (Abbott Laboratories, Abbott Park, IL, USA). Vitamin D status was categorized as deficient (<20 ng/mL), insufficient (21-29 ng/mL), or sufficient (30-100 ng/mL). Statistical analyses included chi-square tests, ANOVA, and multivariate logistic regression.

Results: The mean age of participants was 63.2 ± 6.7 years, and 56.7% were female. Overweight (35%) and obesity (23.6%) were common. Vitamin D deficiency was present in 51.1% of patients, and insufficiency in 18.9%. Radiographically, K-L grade 2 (32.2%) and grade 3 (31.2%) were the most frequent findings. Gender was significantly associated with vitamin D status (p = 0.026), and hypocalcemia was more prevalent among vitamin D-deficient individuals (p = 0.028). However, vitamin D status, vitamin D concentration, age, BMI, and disease duration showed no independent association with radiographic osteoarthritis (OA) severity in multivariate analysis.

Conclusions: Vitamin D deficiency was highly prevalent in this cohort; however, serum vitamin D levels did not independently predict radiographic severity of KOA. These findings suggest that mechanical and age-related factors may influence structural joint degeneration more strongly than vitamin D status alone. We need larger, multicenter longitudinal studies to elucidate the clinical significance of vitamin D in the progression of KOA in Bangladesh.

## Introduction

Osteoarthritis (OA) is a multifactorial degenerative joint disease and a major global cause of pain and disability, significantly reducing quality of life [1,2]. Affecting over 7% of the world’s population, its prevalence is projected to increase by 60% to 100% by 2050 [3,4]. OA most commonly involves the knee and hip, where symptoms such as pain and swelling arise due to genetic susceptibility, biomechanical stress, and joint structural changes, with obesity, trauma, and occupational loading serving as key risk factors [4-6].

Knee OA (KOA) represents the most common and disabling form of the disease, with the highest global incidence [7]. Its pathophysiology involves multiple joint tissues, including articular cartilage, subchondral bone, synovium, and meniscus [8]. Moderate to severe KOA pain is strongly associated with reduced functionality and mobility, limiting daily activities [9]. Radiographic evaluation of KOA generally depends on the identification of osteophytes and joint-space narrowing, and it continues to be the predominant approach for assessing the severity of structural disease [10]. Nonetheless, symptomatic manifestations such as pain and functional impairment do not consistently align with imaging results; certain individuals may endure considerable knee pain without radiographic evidence of OA, while others may exhibit significant structural deterioration despite being asymptomatic [11]. This discrepancy underscores the constraints of symptom-based outcomes, which are affected by psychosocial factors, central sensitization, and individual pain perception, hence endorsing the utilization of radiographic grading as a more objective indicator of illness severity.

Considering the complex characteristics of KOA, modifiable biological variables have garnered heightened scientific focus. Recent evidence indicates that sufficient vitamin D is crucial for musculoskeletal function [12,13] and cartilage-bone metabolism [14]. Conversely, deficiency has been linked to diminished skeletal muscle vitamin D receptor expression and muscle mass loss, especially in older adults [12]. Nonetheless, the correlation between vitamin D levels and the severity of KOA continues to be debated. Numerous studies indicate enhancements in pain and functionality subsequent to vitamin D supplementation, although others have shown no substantial advantages on clinical outcomes or the structural advancement of the disease [15-17]. Vitamin D deficiency is widespread in South Asian communities, especially in Bangladesh [18], rendering its potential correlation with radiographic KOA a significant public health concern.

This study aimed to assess the association between serum vitamin D levels and radiographic severity of KOA, as evaluated using the Kellgren-Lawrence (K-L) grading system, and to explore its relationship with selected demographic, clinical, and biochemical factors among patients attending a tertiary care center in Bangladesh.

## Materials and methods

This cross-sectional analytic study was conducted in the Department of Physical Medicine and Rehabilitation at Bangladesh Medical University (BMU), Dhaka, Bangladesh, from June 2021 to June 2022, following approval from the institutional ethics committee. Patients aged 50 years and above presenting with knee pain to the outpatient department were screened consecutively, and those fulfilling the diagnostic criteria for primary KOA according to the American College of Rheumatology (ACR) [19] were considered eligible for inclusion.

A purposive sampling technique was employed to enroll participants who met the predefined inclusion criteria within the study period. This approach ensured inclusion of clinically and radiographically confirmed cases; however, it may introduce selection bias and limit generalizability beyond the study population. A total of 90 participants who provided informed written consent were included. Patients with inflammatory arthritis, neuropathy, recent knee trauma, previous knee surgery, intra-articular procedures within the preceding six months, underlying malignancy, intra-articular tumors, severe anemia, pregnancy, or use of medications known to affect vitamin D metabolism were excluded to minimize potential confounding.

Demographic characteristics, clinical history, and symptom profiles were recorded using a structured data collection sheet. Weight-bearing anteroposterior and lateral radiographs of the affected knees were obtained from the Department of Radiology and Imaging. Radiographic osteoarthritis severity was graded using the K-L classification system [20]. Radiographic assessment was performed by a single experienced radiologist who was blinded to participants’ serum vitamin D and biochemical results to reduce observer bias. Formal inter- or intra-observer reliability testing was not performed and is acknowledged as a study limitation. For analytical purposes, K-L grades 1-2 were categorized as mild KOA, while grades 3-4 were classified as severe KOA.

BMI was calculated for all participants and categorized according to the World Health Organization (WHO) classification [21]. Serum 25-hydroxyvitamin D levels were measured in the Department of Biochemistry using the Abbott Architect chemiluminescent microparticle immunoassay (CMIA) (Abbott Laboratories, Abbott Park, IL, USA), a validated quantitative method for assessing total vitamin D concentration. The laboratory followed internal quality control procedures in accordance with manufacturer and institutional standards. Vitamin D status was defined as deficient (<20 ng/mL), insufficient (21-29 ng/mL), or sufficient (30-100 ng/mL) based on established guidelines [22]. Normal total serum calcium was defined as 2.1-2.6 mmol/L [23].

Potential confounding variables, including age, BMI, and duration of KOA, were recorded and considered during statistical analysis. Although physical activity level and sun exposure are known to influence vitamin D status, these variables were not quantitatively assessed and are acknowledged as potential residual confounders.

As this was a cross-sectional analytic study, no follow-up assessments were performed, and treatment outcomes were not evaluated. Participants received routine standard-of-care management as per departmental protocol, which was not modified for study purposes.

Ethical approval was obtained from the Institutional Review Board of BMU (Approval No. BSMMU/2019/7773). Participation was voluntary, confidentiality was maintained, and participants retained the right to withdraw at any stage without penalty. No financial incentives were provided, and no conflicts of interest were declared.

Data completeness was verified prior to analysis. Statistical analyses were performed using IBM SPSS Statistics software, version 24 (IBM Corp., Armonk, NY, USA). Continuous variables were expressed as mean ± standard deviation and compared using one-way ANOVA, where appropriate. Categorical variables were presented as frequencies and percentages, and associations were assessed using the chi-square test. Multivariate logistic regression analysis was performed to identify independent predictors of radiographic KOA severity. A p-value < 0.05 was considered statistically significant.

## Results

The socio-demographic profile showed that 52.2% of the participants were aged 50-65 years, while 47.8% were older than 65 years, with a mean age of 63.2 ± 6.7 years. Females constituted 56.7% of the study population. Regarding occupation, 26.7% were homemakers and 28.9% were unemployed, indicating a predominantly non-working or low-activity population, as shown in Table 1.

**Table 1:** Socio-demographic characteristics of the study paticipants (n=90)

Variables	Frequency (n)	Percentage (%)
Age 50-65 years	47	52.2
Age >65 years	43	47.8
Age (years) Mean ± SD	63.2 ± 6.7	—
Female	51	56.7
Male	39	43.3
Homemaker	24	26.7
Service	15	16.7
Business	17	18.9
Skilled worker	8	8.8
Unemployed	26	28.9

**Figure 1:**
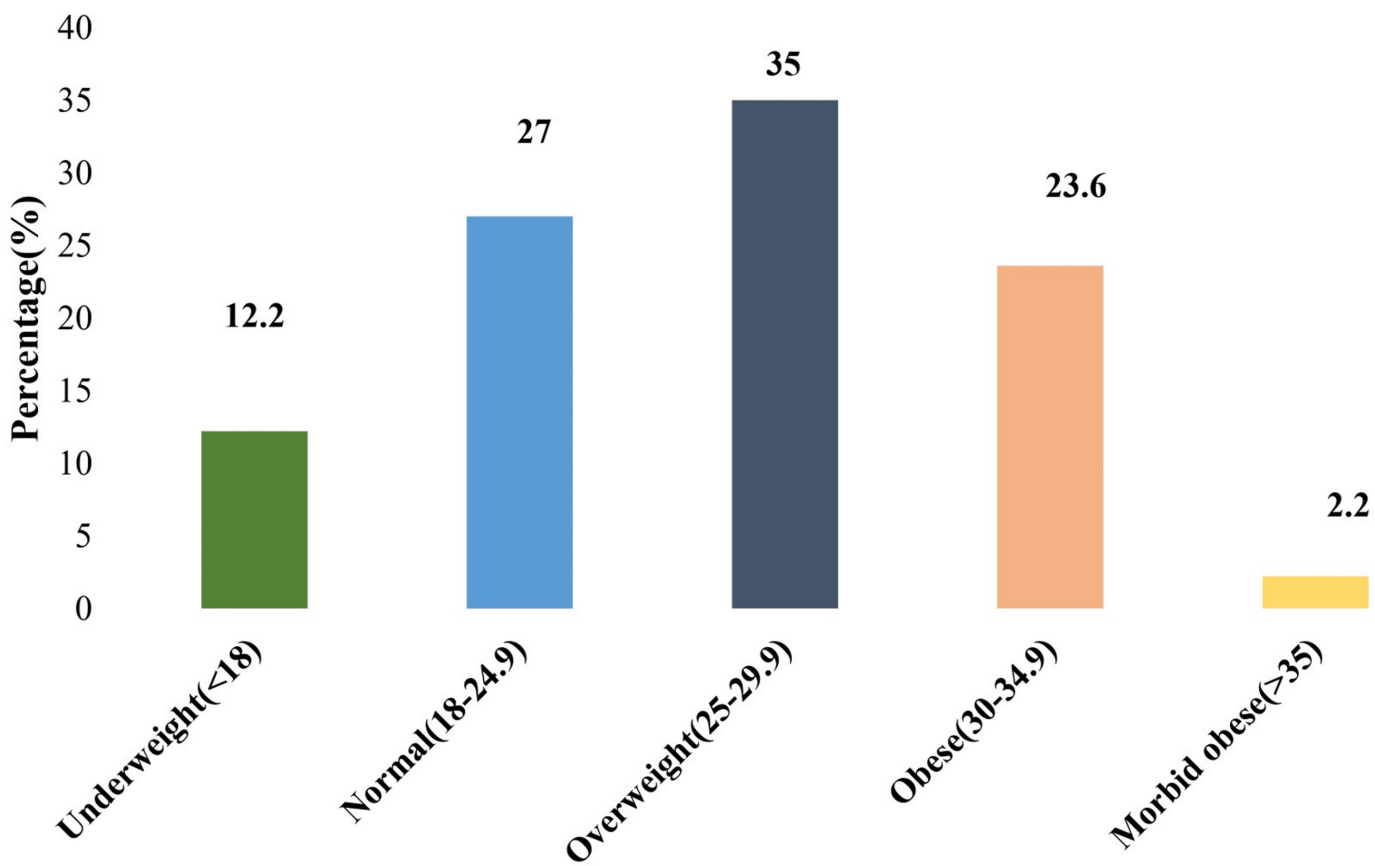
Distribution of BMI of the study participants (n=90) BMI categories were defined according to the WHO classification [21]; BMI measured in kg/m^2^.

The mean BMI of the participants was 26 kg/m². Overall, 35% of participants were overweight, and 23.6% were obese, demonstrating a high prevalence of excess body weight among patients with KOA, as shown in Figure 1. 

Radiographic evaluation revealed that K-L grade 2 (32.2%) and grade 3 (31.2%) were the most frequently observed findings, while 22.2% of participants showed no radiographic osteoarthritis. Disease duration exceeded six months in 43.3% of cases. Based on radiographic severity categories, 42.2% of participants had mild OA (K-L grades 1-2) and 35.6% had severe OA (K-L grades 3-4), indicating that a substantial proportion presented with advanced structural disease, as shown in Table 2.

**Table 2:** Characteristics of knee osteoarthritis among the study participants (n=90) The Kellgren–Lawrence (K-L) grading system was used for radiographic classification [20].

Variables	Category	Frequency (n)	Percentage (%)
Radiographic findings	Normal	20	22.2
Radiographic findings	K–L Scale Grade 1	9	10
Radiographic findings	K–L Scale Grade 2	29	32.2
Radiographic findings	K–L Scale Grade 3	28	31.2
Radiographic findings	K–L Scale Grade 4	4	4.4
Duration of disease	<1 month	19	21.1
Duration of disease	1 to 6 months	32	35.6
Duration of disease	>6 months	39	43.3
Severity of knee osteoarthritis	Normal	20	22.2
Severity of knee osteoarthritis	Mild	38	42.2
Severity of knee osteoarthritis	Severe	32	35.6

**Figure 2:**
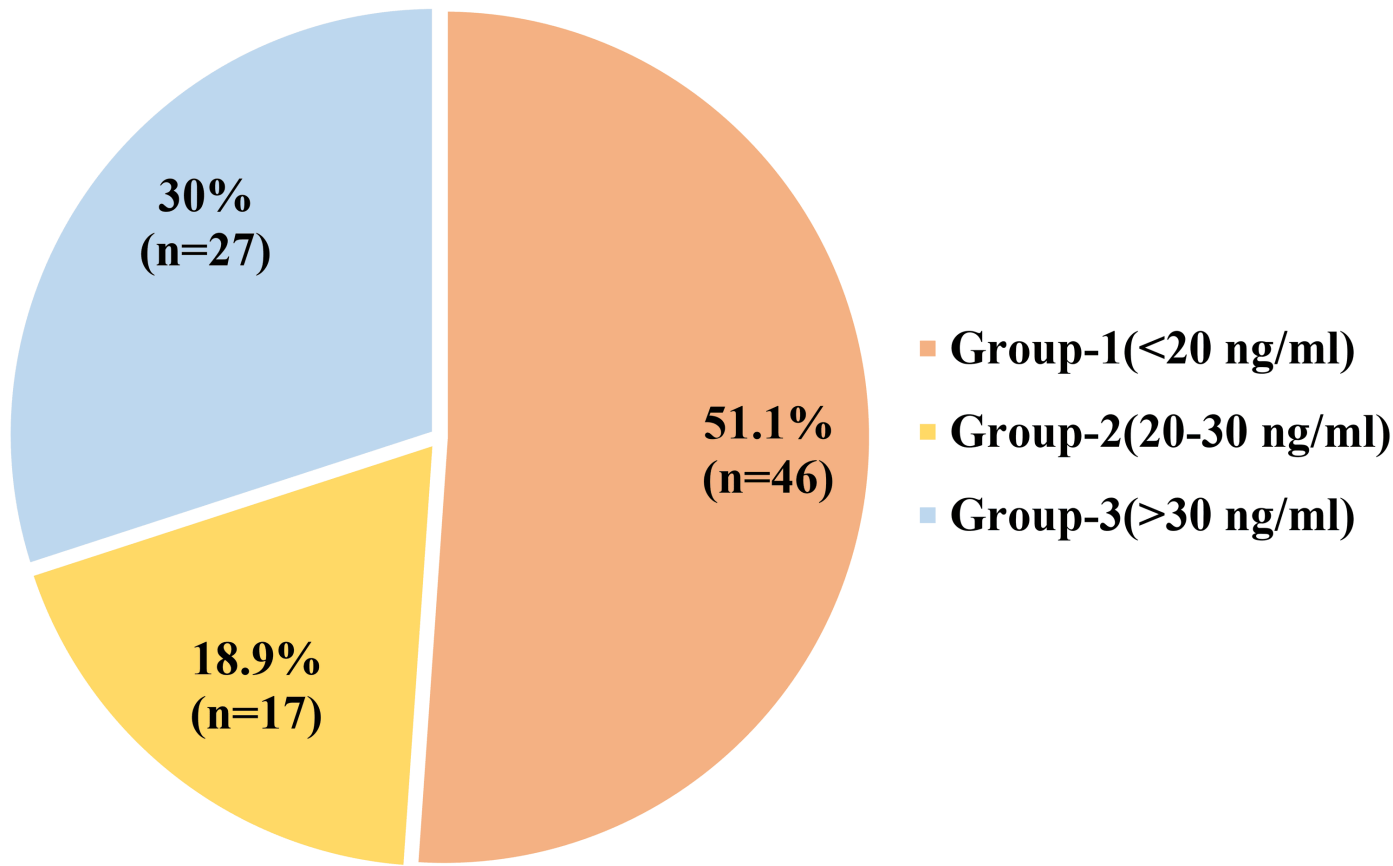
Distribution of vitamin D concentration of the study participants (n=90) Vitamin D categories were defined according to the Endocrine Society guideline cut-offs [22].

Assessment of vitamin D status showed that 51.1% of participants were vitamin D deficient, 18.9% were insufficient, and 30% had sufficient vitamin D levels, indicating a high burden of hypovitaminosis D in the study population, as shown in Figure 2.

Gender demonstrated a statistically significant association with vitamin D status (p = 0.026), with vitamin D deficiency being more common among females (60.9%) than males (39.1%). Although the mean BMI was highest in the vitamin D-deficient group (27.0 ± 3.59 kg/m²), the difference across vitamin D categories was not statistically significant (p = 0.089). Similarly, radiographic grades (p = 0.098) and disease duration (p = 0.057) did not differ significantly across vitamin D categories. In contrast, OA severity showed a statistically significant association with vitamin D status (p = 0.048), with severe OA observed in 45.7% of vitamin D-deficient participants, as shown in Table 3.

**Table 3:** Association of vitamin D deficiency and characteristics of the patients (n=90) **p-value obtained by chi-square test; *p-value obtained by one-way ANOVA; KOA: knee osteoarthiritis The Kellgren–Lawrence (K-L) grading system was used for radiographic classification [20]. Vitamin D categories were defined according to the Endocrine Society guideline cut-offs [22].

Characteristics	Group-1 (Vitamin D Deficiency)	Group-2 (Vitamin D insufficiency)	Group-3 (Vitamin D normal)	Test statistics value (χ² / F)	p-value
	n=46	n=17	n=27		
	n (%)	n (%)	n (%)		
Male	18 (39.1)	4 (23.5)	17 (63)	7.28	0.026**
Female	28 (60.9)	13 (76.5)	10 (37)		
Age (in years), Mean ±SD	62.4±6.39	62.0±7.14	60.2±5.89	3.80	0.062*
BMI (kg/m^2^), Mean ±SD	27.0±3.59	25.8±5.21	24.4±6.09	2.48	0.089*
K-L Scale Grade 1	4 (8.7)	2 (11.8)	3 (11.2)		
K-L Scale Grade 2	11 (23.9)	9 (52.9)	9 (33.3)	14.34	0.098**
K-L Scale Grade 3	19 (41.4)	3 (17.6)	6 (22.2)		
K-L Scale Grade 4	2 (4.3)	2 (11.8)	0 (0)		
Normal	10 (21.7)	1 (5.9)	9 (33.3)		
Duration of KOA (>1month)	16 (34.8)	3 (17.6)	6 (22.3)	7.11	0.057**
Duration of KOA (1-6 months)	8 (17.4)	9 (53)	12 (44.4)		
Duration of KOA (>6 months)	22 (47.8)	5 (29.4)	9 (33.3)		
Severity of KOA (Normal)	10 (21.7)	1 (5.9)	9 (33.3)	10.35	0.048**
Severity of KOA (Mild: K-L Scale Grades 1 & 2)	15 (32.6)	11 (64.7)	12 (44.4)		
Severity of KOA (Severe: K-L Scale Grades 3 & 4)	21 (45.7)	5 (29.4)	6 (22.3)		

**Figure 3:**
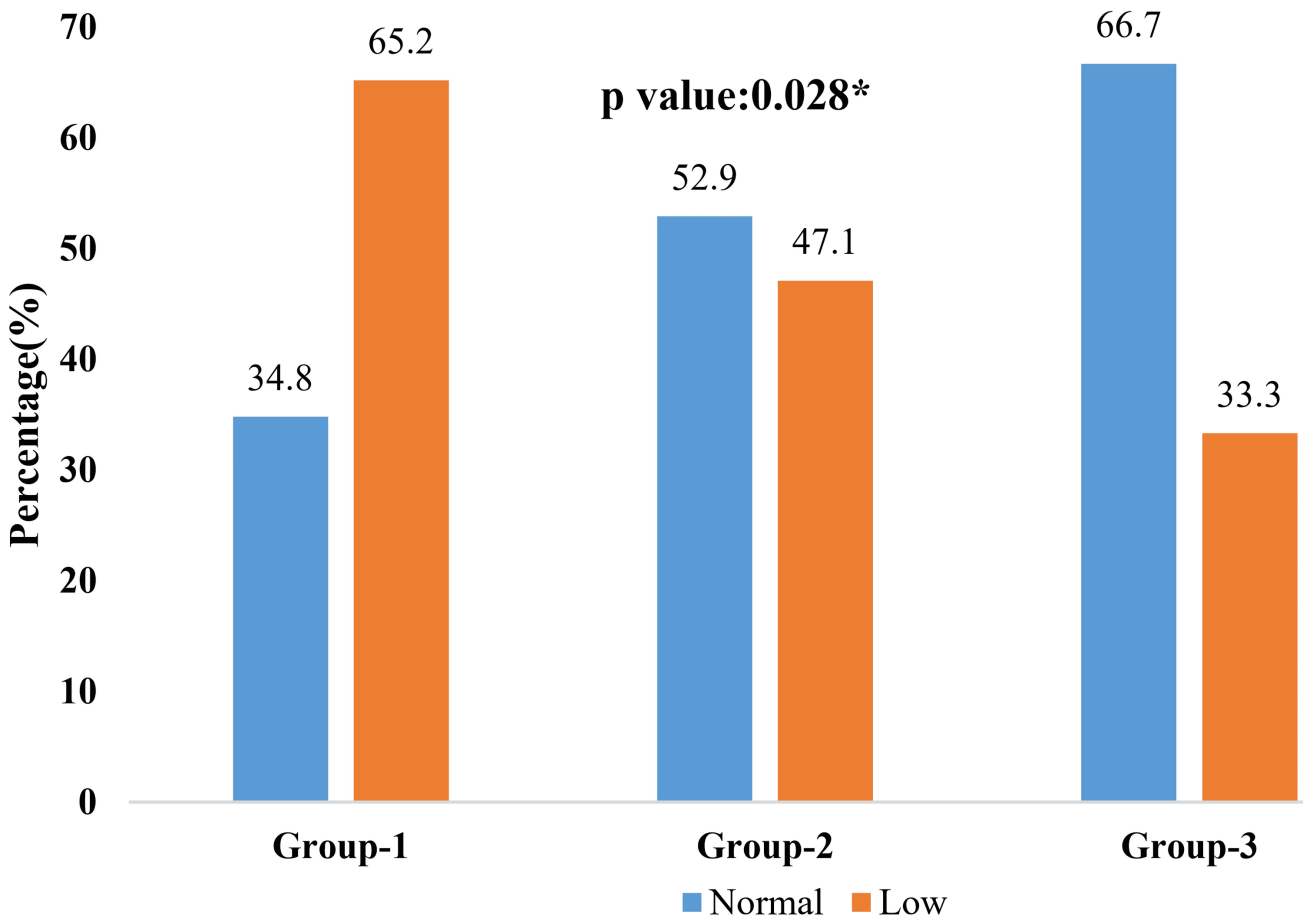
Distribution of calcium level (mmol/l) according to vitamin D concentration among the patients (n=90) *p-value obtained by chi-square test. Group 1: vitamin D deficiency; Group 2: vitamin D insufficiency; and Group 3: normal vitamin D. Vitamin D categories were defined according to the Endocrine Society guideline cut-offs [22]. Normal total serum calcium was defined according to Davidson’s Principles and Practice of Medicine reference ranges [23].

Serum calcium levels differed significantly across vitamin D categories (p = 0.028), with low serum calcium levels observed in 65.2% of vitamin D-deficient participants, indicating a significant biochemical association between vitamin D deficiency and hypocalcemia, as shown in Figure 3.

Multivariate logistic regression analysis showed that none of the evaluated variables, including age, BMI, disease duration, serum vitamin D level, vitamin D category, or serum calcium level, had a statistically significant independent association with radiographic KOA severity (all p > 0.05). Although vitamin D deficiency was associated with higher odds of severe OA (OR 2.37, 95% CI 0.50-11.22) and vitamin D insufficiency with higher odds of mild OA (OR 5.80, 95% CI 0.60-55.97), these associations were not statistically significant after adjustment for potential confounders, as shown in Table 4.

**Table 4:** Multivariate logistic regression showing association of radiographic knee osteoarthritis with risk factors *reference category: normal, no radiographic changes; ** reference category: normal vitamin D level; ***reference category: normal serum calcium level The Kellgren–Lawrence (K-L) grading system was used for radiographic classification [20]. Vitamin D categories were defined according to the Endocrine Society guideline cut-offs [22].

Variables	Mild knee osteoarthritis (KL grade-1 and KL grade-2)* n=38		Severe osteoarthritis (K-L Grade 3 and K-L Grade 4)* n=32	
	OR (95% CI)	p-value	OR (95% CI)	p-value
Age	1.08 (0.99-1.17)	0.083	1.01 (0.93-1.10)	0.745
BMI (kg/m^2^)	0.97 (0.87-1.09)	0.673	1.12 (0.98-1.27)	0.080
Duration of disease (months)	0.94 (0.82-1.08)	0.398	0.99 (0.86-1.14)	0.927
Vitamin D level (ng/ml)	1.02 (0.95-1.08)	0.522	1.00 (0.93-1.08)	0.956
Vitamin D (deficiency) **	0.87 (0.24-3.18)	0.835	2.37 (0.50-11.22)	0.278
Vitamin D (insufficiency) **	5.80 (0.60-55.97)	0.128	2.77 (0.20-38.82)	0.451
Total calcium (mmol/l)***	1.10 (0.37-3.26)	0.864	1.22 (0.40-3.75)	0.726

## Discussion

This study examined the association between serum vitamin D status and radiographic KOA in an older outpatient population. Most participants were above 60 years of age and predominantly female, with a large proportion being homemakers or unemployed, reflecting a low-activity lifestyle that may predispose them to musculoskeletal decline. These findings are consistent with Swailem et al. (2025), who reported a high prevalence of vitamin D deficiency among older women with KOA [24]. Overweight and obesity were also highly prevalent in this cohort, supporting previous evidence that excess body weight is an important mechanical contributor to knee joint degeneration [25,26].

Vitamin D deficiency emerged as a common biochemical abnormality and aligns with prior evidence suggesting that low vitamin D levels may influence OA-related molecular and musculoskeletal pathways, including reduced GDF-5 expression [27]. Female participants were disproportionately vitamin D deficient, consistent with regional data highlighting the vulnerability of South Asian women due to limited sunlight exposure and dietary insufficiency [18]. Although radiographic severity differed across vitamin D categories in unadjusted analyses, these associations did not remain statistically significant after multivariate adjustment. This finding is in agreement with reports such as the one by Mermerci Başkan et al. (2017), which found no definitive association between serum vitamin D levels and radiographic KOA severity [28], despite other studies suggesting links with pain or functional outcomes rather than structural progression [29,30].

Hypocalcemia was more prevalent among participants with vitamin D deficiency, reinforcing a biochemical association between vitamin D status and musculoskeletal metabolism. However, the lack of an independent association between serum vitamin D levels and radiographic severity suggests that structural joint degeneration in KOA may be more strongly influenced by factors such as obesity, aging-related cartilage loss, reduced physical activity, and cumulative mechanical loading than by vitamin D status alone.

Importantly, interpretation of these findings must account for several methodological constraints inherent to the study design. As a cross-sectional analysis, this study does not allow causal or temporal inferences, and it remains unclear whether low vitamin D levels contribute to greater OA severity or whether advanced disease and reduced mobility lead to reduced vitamin D levels through diminished sunlight exposure and physical activity. The use of purposive sampling and single-center recruitment may have introduced selection bias and limited the generalizability of the findings beyond the study population. Additionally, although radiographic grading provides an objective measure of structural disease, it may not fully reflect symptomatic burden or functional impairment. Potential confounding factors, including dietary habits, sun exposure, physical activity levels, and comorbid conditions, were not quantitatively assessed and may have resulted in residual confounding. Furthermore, while radiographic assessments were performed by an experienced radiologist and standardized laboratory assays were used, formal inter- or intra-observer reliability testing and external laboratory calibration were not conducted, which may have introduced observer- or laboratory-related variability.

Despite these methodological considerations, the high prevalence of vitamin D deficiency and hypocalcemia observed in this population has meaningful clinical implications. Screening and correction of vitamin D deficiency in older adults with knee pain may improve musculoskeletal performance, balance, and fall risk, even if radiographic progression remains unaffected. Future multicenter studies with larger sample sizes and longitudinal follow-up are warranted to clarify the temporal and clinical significance of vitamin D in the progression of KOA in Bangladesh and similar settings.

## Conclusions

In this study, vitamin D deficiency was highly prevalent among older adults presenting with knee pain; however, serum vitamin D levels showed no independent association with radiographic KOA severity. Given the cross-sectional design, causal relationships cannot be inferred, and the findings should be interpreted as associations rather than evidence of causation. Structural joint degeneration in KOA may be more strongly related to mechanical loading, aging, and obesity than to vitamin D status alone. Larger, multicenter longitudinal studies from Bangladesh and similar settings are needed to clarify the temporal and clinical significance of vitamin D in KOA progression.
